# Effectiveness of UK-based support interventions and services aimed at adults who have experienced or used domestic and sexual violence and abuse: a systematic review and meta-analysis

**DOI:** 10.1186/s12889-025-21891-5

**Published:** 2025-03-14

**Authors:** Sophie Carlisle, Annie Bunce, Matthew Prina, Sally McManus, Estela Barbosa, Gene Feder, Natalia V. Lewis

**Affiliations:** 1 Department of Health Service and Population Research, Institute of Psychiatry, Psychology and Neuroscience, King’s College London, De Crespigny Park, London, UK; 2 Health Innovation East Midlands, University of Nottingham- Jubilee Campus, Nottingham, NG7 2TU UK; 3 Violence and Society Centre, City St. George’s, University of London, London, UK; 4 Faculty of Medical Sciences, Population Health Sciences Institute, Newcastle University, Newcastle, NE4 5PL UK; 5 National Centre for Social Research, London, UK; 6 Centre for Academic Primary Care, Bristol Medical School, University of Bristol, Bristol, BS8 2PS UK

**Keywords:** Domestic abuse, Sexual violence, Services, Interventions, Safety, Wellbeing, Systematic review

## Abstract

**Background:**

Domestic and sexual violence and abuse (DSVA) is prevalent and harmful. There are a range of support services and interventions available to those affected by it, but evidence of their effectiveness is uncertain. We synthesised evidence on the effectiveness of UK-based interventions and services for DSVA.

**Methods:**

We conducted a systematic review and, where possible, meta-analysis. We searched MEDLINE, EMBASE, PsycINFO, Social Policy and Practice, ASSIA, IBSS, Sociological abstracts, SSCI and grey literature sources for publications published from inception to July 2023. We included randomised controlled trials, non-randomised comparative studies, pre-post studies, and service evaluations of support interventions or services for adults who had experienced or perpetrated DSVA. The intervention typology and selection of outcomes was determined based on co-production with stakeholders. The quality of the studies was assessed independently by two reviewers. Where meta-analysis was not possible, we synthesized studies with vote counting based on the direction of effect.

**Results:**

Twenty-nine UK-based studies were included: 11 on advocacy, five on outreach, six on psychological interventions or services for victims-survivors, and six on perpetrator programmes. Meta-analyses showed benefits, with 58.7% (95% CI 53.6, 63.8) of advocacy and 46.2% (95% CI 39.1, 53.3) of outreach intervention and service participants reporting cessation of abuse at case closure. Vote counting was performed for psychological support interventions and perpetrator programmes, and showed positive effects on self-esteem and attitudes towards sexual offending. Most studies had a high risk of bias.

**Conclusions:**

There appear to be benefits of UK-based advocacy and outreach services, psychological support interventions, and perpetrator programmes. However, risk of bias and methodological heterogeneity means that there is uncertainty regarding the estimated effects. There is need for more robust research, and a co-produced core-outcome set to facilitate future research in this field.

**Trial registration:**

PROSPERO (CRD42022339739).

**Supplementary Information:**

The online version contains supplementary material available at 10.1186/s12889-025-21891-5.

## Background

Domestic and sexual violence and abuse (DSVA) refers to physical, sexual, emotional, and any other form of violence and abuse from a current or former partner or family member, and sexual violence and abuse from non-partners. DSVA is prevalent globally, including in the UK. In the year ending March 2022 over 1.5 million domestic abuse-related incidents and crimes were recorded by the police [1], and a further 193,000 sexual offences were recorded in the same period [2]. An estimated 10.4 million people aged 16 years and over have experienced domestic abuse [1], while 7.9 million have experienced sexual assault in England and Wales since the age of 16 [3]. These figures are likely to be underestimates, with fewer than 24% of domestic abuse-related crimes being reported to police [4], and five in six women who are raped not reporting [5]. Underreporting experiences of violence in surveys such as the Crime Survey for England and Wales can result from social stigma [6], or from fear where victim-survivors are still living with someone who uses violence, and be influenced by the survey framing (e.g., whether focused on health or crime) [7].

The impacts of DSVA are wide ranging, for both individuals and society. DSVA damages both physical [8–14] and mental health [7, 15–19], financial stability, relationships, and housing security [20–22]. Societal costs include strain on the criminal justice system, health and social services, and police. For instance, police in England and Wales receive an estimated 100 calls per hour relating to domestic abuse [23], and the total police costs associated with domestic abuse incidents are estimated at £999 million [24]. The overall cost of domestic abuse over a one-year period (March 2016–2017), including costs to victims, the economy, health services, police, government and charities, has been estimated at £66 billion [24]. Further, the economic and social cost of rape and other sexual offences for 2015–2016 has been estimated at £12.2 billion [25].

Due to the high cost of DSVA, developing effective responses is crucial. It is internationally recognised that preventing the recurrence of DSVA and preventing or limiting its impacts means changing social norms, attitudes and behaviours that underpin violence, which requires intervention at individual, relationship, community/organisational and societal levels [26]. Interventions to prevent revictimisation and perpetration focus on addressing these root causes, as well as risk and protective factors known to be associated with violence, by providing remedy and support to victim-survivors to empower them to regain control of their lives, and holding perpetrators accountable whilst offering them meaningful opportunities to change [27]. Whilst the theory(s) underpinning DSVA interventions differ according to their specific aims and remits, most draw upon a combination of patriarchal/feminist, psychopathological, intersectional and systems-level theories and principles [28, 29].

In the UK, there are a range of support services and interventions for people who have experienced DSVA, including refuges, advocacy such as Independent Domestic Violence Advisors (IDVAs), referral, outreach, and helplines. These are often provided by the Voluntary and Community Sector (VCS), although may also be located in the public or private sectors. The specific aims of each type of service and intervention vary, as do the specific type(s) of support offered, be that practical (e.g., housing, financial support), psychological (e.g., increased coping and resilience, space to process trauma), or informational (e.g., about other services, options, and next steps). While the specific mechanisms underlying the benefits of such support for those accessing them are unclear and vary between individuals, one potential mechanism is that accessing these types of support and resources may improve mental health, wellbeing, and feelings of empowerment. In turn, this may facilitate those experiencing DSVA to be in a better position to achieve their own goals and live a life free from abuse [30, 31]. Domestic Abuse Perpetrator Programmes (DVPPs/ DAPPs; hereon referred to as perpetrator programmes) are another type of support service that aims to keep survivors safe and hold perpetrators accountable [32]. Rehabilitative work with domestic violence perpetrators exists largely in the form of behavioural change “treatment” interventions, based on the principle that men must take responsibility for their abusive behaviour and that such behaviour can be unlearned [33]. Perpetrator programmes provide various services and information to clients, including skills training, cognitive behavioural therapy (CBT), motivational interviewing, psychoeducational interventions and work around social learning, power and control [34]. As well as working with perpetrators on a one-to-one or group basis, some perpetrator programmes often work with partners and/or families as well. UK evaluations have employed a wide range of outcome measures, including reductions in or cessation of abusive behaviour, attitudes and beliefs on gender, women and violence, levels of and resilience to repeat victimisation, quality of life (of both the perpetrator and the victim/partner), feelings of safety and well-being of women/partners (and their children), and levels of parenting stress [33, 35].

Existing systematic reviews of DSVA services and interventions [36–39] and perpetrator programmes [33, 40] are limited in that: (1) they focus on a single type of support intervention or service and therefore cannot make comparisons across service types; (2) many have not performed comprehensive grey literature searches or included stakeholder advisory groups and therefore may not be accurate reflections of the full picture, a particular drawback given that much of the evidence-base in the field of DSVA is not published in peer-reviewed formats; and (3) they are not directly applicable to the UK service and policy context.

One problem facing syntheses of evidence in this field is the wide-ranging outcomes used to assess effectiveness. Our recent scoping review identified 426 outcomes across 80 studies, with only 46.9% used in more than one evaluation [41]. As a result of this scoping review, we recommended the development of a core outcomes set, co-developed with funders, service providers and people with lived experience, so that a more cohesive and relevant evidence base can be built. For this review, we use findings from our scoping review which identified the most commonly reported outcomes, including outcomes relating to safety and wellbeing, together with stakeholder consultation, to inform and direct the focus of the review, and to best synthesise the current evidence base.

Our aim was to review the peer reviewed and grey literature to identify studies that assessed the effectiveness of support interventions and services for people who have experienced DSVA. This review was conducted as part of a programme of research undertaken by the VISION Consortium aiming to reduce violence and health inequalities through better measurement and integration of data.

### Review question

How effective are UK-based support interventions and services (targeted at adults of any gender who have experienced or used DSVA) at improving safety and wellbeing?

## Methods

### Protocol and registration

The review follows the Preferred Reporting Items for Systematic Reviews and Meta-Analysis (PRISMA) [42] checklist and Synthesis without meta-analysis (SWiM) reporting guidelines [43] (Additional file 1). The protocol for the review has been registered on Prospero: CRD42022339739.

### Deviations from the protocol

The review largely adhered to the published protocol. However, one exception was the categorisation of interventions and services. In the protocol, we proposed intervention and service categories that included combined outreach and IDVAs under the umbrella term ‘community outreach’. However, during the process of the review, this was amended in line with a series of reports published by SafeLives, a UK-based domestic abuse charity that provides frontline services and collects and publishes national data and evaluation reports. These SafeLives Insights reports provide data from the largest dataset on domestic abuse in the UK, gathered from services working with victim-survivors of domestic abuse. On the basis of these reports, which provide data separately for outreach and IDVA services, we also separated these into two forms of intervention and services.

Additionally, we originally aimed to describe the included studies according to the TIDieR framework [44], however ultimately opted not to as many of the studies described services rather than traditional interventions, which did not map well onto the TIDieR framework.

### Eligibility criteria

#### Population

Adults who have experienced DSVA or who have perpetrated DSVA. Adults were defined as those aged 16 years or older, consistent with the National Institute for Health and Care Excellence quality standard for domestic violence and abuse. DSVA was defined according to the UK cross-governmental definition of domestic violence and abuse (DVA) (2013) [45], the Domestic Abuse Act 2021 [46], the Istanbul Convention (Article 36) [47], the World Health Organisation definition of sexual violence and abuse [48], and the Rome Statute of the International Criminal Court’s (ICC) Elements of Crimes (2013) [49]. The distinctions and overlaps between these definitions were discussed in the review protocol [50]. While this review uses the term ‘people who have experienced DSVA’, it should be noted that there are different terminology preferences between organisations within the VCS, therefore this may also be used interchangeably to mean victims of DSVA, survivors of DSVA, and victim-survivors. Similarly, while this review refers to ‘perpetrators of DSVA’, this term has been contested by some who prefer the term ‘people who use violence’. No limit was placed on time since the experience of DSVA, so long as participants accessed the intervention or service as an adult.

#### Interventions/services and outcomes

The specific forms of interventions and services (hereafter referred to as ‘interventions’ only) included in this review were determined by a two-stage process. Initially, any outcome relating to safety or wellbeing and any form of support intervention meeting the following criteria was included:

- Studies of any secondary or tertiary prevention support interventions were eligible for inclusion. Primary prevention was not included as these target people who have not yet experienced violence.
- Entry to the intervention had to be determined by the experience of DSVA.
- There was no restriction placed on the format or duration of the intervention.
- Interventions that are not primarily aimed at DSVA were excluded.
- Perpetrator programmes were included as they are another form of intervention that may be effective in reducing DSVA and improving outcomes for people who have experienced DSVA.
- Outcome data had to be reported for two or more time-points and/or for two or more groups, so that cause and effect could be inferred.

Following consultation with stakeholders (see the stakeholder consultation section for more details) and according to the results of our scoping review [51], it was agreed that only the most commonly reported outcome for each category of intervention would be included (or outcome*s*, if the most commonly reported outcome was tied between more than one). Additionally, outcomes (and therefore interventions) would only be included if the most common outcome for that category of intervention was reported by at least three studies, to allow for meta-analysis. As a result, four types of interventions and four distinct outcomes were included in the review:

Victim-survivor interventions:

- Advocacy: Cessation of abuse according to the Severity of Abuse Grid
- Outreach: Cessation of abuse according to the Severity of Abuse Grid
- Psychological support: Self-esteem according to the Rosenberg Self Esteem Scale

Perpetrator programmes:

- Balanced Inventory of Desirable Reporting (BIDR); Questionnaire on Attitudes Consistent with Sexual Offending (QACSO)

#### Comparator

Where applicable, comparators could be another intervention, usual care, no support intervention or wait-list controls. For uncontrolled before and after studies, the comparison was the change from pre- to post-intervention.

#### Study designs

Any type of interventional study reporting outcomes at two or more time-points and/or making comparisons between two groups, including randomised controlled trials (RCTs), non-randomised comparative trials, and uncontrolled before and after studies were included. Cross-sectional studies, case control designs, qualitative studies and studies that were descriptive only and did not provide data on effectiveness were excluded.

#### Setting

Any UK setting was included.

#### Other criteria

Given the focus of the review on the UK setting, only English language reports were included. There was no restriction in terms of date.

### Information sources and search strategy

Searches of the following electronic databases of peer-reviewed articles were conducted to identify potentially eligible studies: MEDLINE, EMBASE, PsycINFO, Social Policy and Practice, ASSIA, IBSS, Sociological abstracts, and SSCI. Key search terms included terms relating to DSVA (e.g., “domestic violence”, “partner”, “sexual violence”), specialist support services and interventions (e.g., “specialist service”, “support”, "outreach", “refuge”), and the UK (e.g., “United Kingdom”, “England”, “Wales”, “Scotland”, “London”). Terms were combined using Boolean operators.

A comprehensive grey literature search was also conducted comprising three strategies. Four electronic grey literature databases were searched: National Grey Literature Collection, EThOS, Social Care Online, and the Violence Against Women Network, using a simplified version of the previous search strategy. Search terms included “domestic violence”, “sexual violence”, “service”, “support”, and “intervention”. A call for evidence was also circulated via email to 295 local and national DSVA services and organisations and relevant research networks to request any service evaluations or reports relevant to the review questions and meeting the inclusion criteria to be shared. Contacts were emailed again if there was no initial response after two weeks. Finally, websites of relevant UK-based DSVA organisations were searched for relevant reports, research and publications. Where there were numerous pages of potentially relevant results, only the first five pages were assessed. For websites with a search function, the following terms were searched: “Service”, “Evaluation”, “Intervention”, and “Report”. Both the peer reviewed and grey literature searches were conducted on 21st June 2022 and updated on 5th July 2023.

Backwards and forwards citation tracking was carried out for all included studies, and reference lists of identified and relevant systematic reviews were also checked to identify further potentially relevant studies. See Additional file 2 for an example of the search strategy used for one peer-reviewed and one grey literature database.

### Selection of studies

The process for the selection of studies varied according to the method of identification. All records identified from peer reviewed and grey literature databases were exported into Endnote. All reports obtained from the call for evidence were manually added to the same Endnote Library. Finally, rather than manually adding all reports identified on individual websites, titles and abstracts or descriptions of reports were assessed according to the inclusion and exclusion criteria, and only those deemed potentially relevant were downloaded and manually added to the Endnote library. Duplicates were then removed. The de-duplicated records were uploaded into Rayyan [52], and all were then screened by title and abstract against the inclusion and exclusion criteria for possible inclusion. Where there were multiple reports from the same study, such as a protocol or appendices, the primary report was identified, and additional reports were labelled as subsidiary and given the same study identifier. Thus, the unit of analysis for the review was the study, not each individual report. Reports that appeared to satisfy the eligibility criteria based on titles and abstracts, or where it was unclear, then underwent a full text screening. This was primarily done by one reviewer, with a second reviewer independently screening 20% of titles and abstracts and then full texts. Disagreements between reviewers were resolved by discussion, or through discussion with a third reviewer until consensus was reached.

### Data extraction

A piloted data extraction spreadsheet was used to extract and record information from each included study. This included basic study information, such as authors, date, study design, and funding, information about the setting, participant details, intervention details including comparator groups where appropriate, the reported outcomes and results. Where there were multiple reports from the same study, relevant data from all reports were extracted into a single entry. Data extraction was completed by one reviewer, and independently checked by a second. Any disagreements were resolved through discussion, with a third reviewer involved where discussions could not be resolved. Where data were missing, corresponding authors were contacted and asked to supply said data.

### Risk of bias

All studies underwent a risk of bias assessment. Randomised controlled trials were assessed using the Cochrane Collaboration RoB2 tool [53]. Non-randomised comparative studies were assessed using the Cochrane Collaboration ROBINS-I tool [54]. Non-controlled before and after studies were assessed using an adapted version of the ROBINS-I tool. Finally, grey literature was assessed using the AACDOS tool [55]. Two reviewers independently assessed risk of bias. All disagreements were discussed until a consensus was reached.

### Synthesis of results

We conducted meta-analyses where the data permitted (i.e., there are three or more studies reporting the same outcome measure and sufficient data is reported), and a narrative synthesis for outcomes where meta-analysis was not possible, following the SWiM guidelines [43]. Specifically, we adopted the method of vote counting based on the direction of effect where meta-analysis was not appropriate. The selection of this method was based on the available data in the studies. All studies meeting the inclusion criteria were included in the synthesis, regardless of study design, risk of bias or indirectness. For both meta-analysis and vote counting analysis, studies were grouped according to the type of intervention. This was because the different types of interventions varied in terms of their aims, the type of support provided, and outcomes reported. For vote counting analysis, results are presented using tabular methods, reporting key study characteristics (including study design, sample size and risk of bias), and discussed narratively.

Where appropriate, meta-analysis was conducted using a random effects model in Stata 18. The specific method of meta-analysis varied according to outcome and data type, and study design where applicable. For instance, all but one of the studies reporting the cessation of abuse outcome were uncontrolled before and after studies. There were no statistically robust approaches to meta-analyse dichotomous data for single-group data, and given that at baseline none of the participants would report cessation of abuse, therefore the event rate would be zero, a meta-analysis of proportions was conducted using the post-intervention data only. In effect, this provided both the pooled prevalence of the cessation of abuse, and the change, from pre- to post-intervention. For the three outcomes using continuous data, meta-analyses of change scores were planned using mean change and standard errors, however a combination of insufficient reported data, small study sizes, and inconsistency in how outcomes were utilised ultimately meant that meta-analyses were not appropriate. Results of the meta-analyses are presented using forest plots and discussed narratively.

Levels of heterogeneity were assessed using the I^2^ statistic and Cochran’s Q. Subgroup analyses were planned where heterogeneity was substantial or considerable (defined as I2 = 50–90% and I2 = 75–100%) [56]. Subgroup analyses to investigate heterogeneity included: study design; setting (VCS; private sector; public sector); relationship between the person who has experienced violence and the perpetrator of violence (e.g., (ex)intimate partner; stranger; domestic but not partner; friend/acquaintance; professional; mixed/any); the population the service or intervention is aimed at (e.g., those who have experienced violence; perpetrators of violence; both); type of service or intervention provider (e.g., specialist DSVA; specialist but not DSVA; non-specialist); and type of violence (e.g., primarily DVA focused; primarily sexual violence and abuse (SVA) focused; combined DSVA).

We conducted sensitivity analyses, removing studies that had a high or very high risk of bias and removal of one study at a time, to explore for potential biases. Certainty was assessed using the GRADE framework, which takes into account risk of bias, inconsistency, imprecision, indirectness and publication bias.

### Stakeholder consultation

An advisory stakeholder group comprising professional representatives from six specialist DSVA organisations involved in the delivery, planning, funding or support of specialist DSVA support services in the UK was established as part of the VISION Consortium. The group included representatives from two second-tier (i.e., organisations that support front-line services but do not provide services themselves) domestic abuse organisations, one second-tier organisation for violence against Black and Minority Ethnic women and girls, one domestic abuse organisation that provides a range of front-line services, one service focusing specifically on supporting male victims, working with perpetrators of domestic violence, and working with young people using violence in close relationships, and one service focusing specifically on sexual violence and abuse. The group was recruited by the VISION programme of research to contribute to and co-produce research that improves the understanding of the relationship between violence, health and inequalities and improves data collection for public benefit.

We held two workshops with stakeholders; one in September 2022 and one in June 2023. The two-hour workshops were structured and included a mixture of presentations focused on the systematic review methodologies, and discussions based on open ended questions. During the first workshop, the group inputted to the design of the study protocol and provided insight and context regarding the challenges in measuring the effectiveness of support services in the VCS. Their input resulted in several changes, including broadening the scope of this review to try to identify evidence relating to victim-survivor wellbeing and perpetrator attitudes and behaviour, rather than focusing only on outcomes directly related to violence cessation, to reflect the priorities of the sector.

During the second workshop, stakeholders aided with the interpretation of preliminary data, and helped to shape the analysis approach. For instance, the initial approach to the systematic review was to use the scoping review to identify the five most commonly reported outcomes, and then to work with stakeholders to prioritise these in terms of importance and relevance. However, through discussion with the stakeholders it became clear that it was not appropriate to apply one outcome to each and every type of intervention, as they do not all have the same aims and therefore would not be expected to impact the same outcomes. As a result, the method described in the eligibility criteria section was adopted, whereby the most commonly reported outcomes for each individual intervention were identified.

## Results

### Selection of sources of evidence

**Fig. 1:**
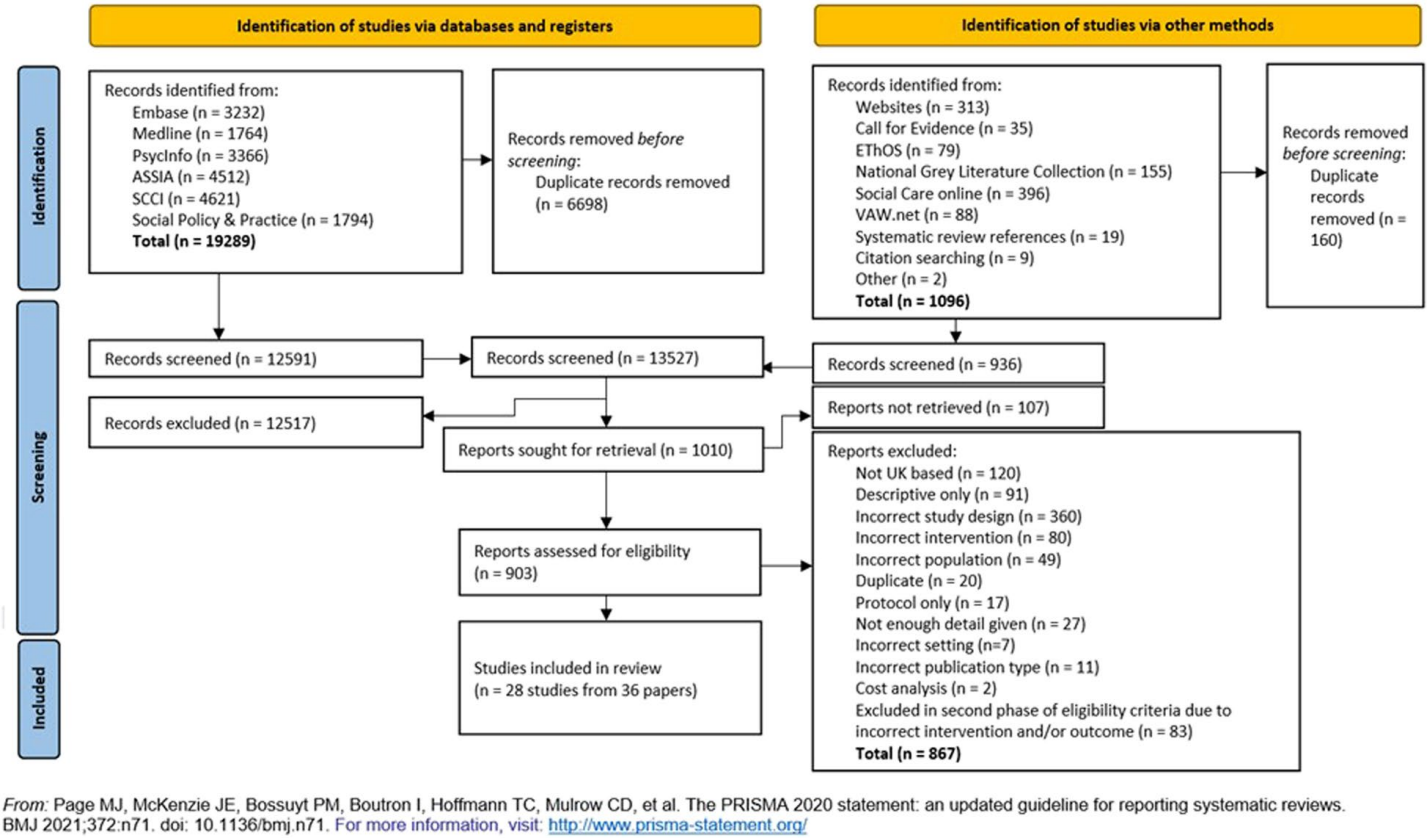
PRISMA flow diagram

The peer reviewed literature search retrieved 19,289 records, and the grey literature search retrieved an additional 1096 records. After duplicates were removed, there was a total of 13,527 records, of which 12,517 were excluded and 903 underwent full text screening. Overall, 28 studies were included from 36 reports [57–92] (Fig. 1).

### Study characteristics

Details of the included studies can be found in Table 1. Of the 28 studies, 23 described interventions for people who have experienced DSVA, while six described programmes aimed at perpetrators of DSVA. The interventions for people who have experienced DSVA involved a total of 42,850 participants, the majority of whom were heterosexual, White British, and predominantly women. A further 246 participants were included in the perpetrator programmes, all of whom were men. Eighteen of the studies focused on DVA only, five focused on adult victims of childhood sexual abuse (CSA), three focused on SVA, and two included multiple forms of abuse. The majority (n = 17) were based in the VCS. Of the 23 studies describing interventions for victim-survivors, ten were produced by SafeLives as part of their Insights outcome measurement reports.

**Table 1:** Details of included studies

Study	Grey lit or peer review	Study design	Sector	Participants	Description of intervention and comparator	Type of abuse	Outcome
**Advocacy**							
CAADA 2012 [56, 92]	Grey lit	Uncontrolled pre-post	VCS	2653 service users engaging with the service • 92% female • 35% aged 21–30 years • 96% heterosexual • 85% White British or Irish	Advocacy (IDVA): 14 domestic abuse services. The most common types of support accessed included safety planning, health and wellbeing advice and support, support with MARAC, and liaison and support with police Duration: Median case length was 2.6 months Comparator: N/A	DVA, SVA	Cessation of abuse at exit compared to intake
Halliwell 2019 [58]	Peer review	Non-randomised comparative	Mixed	4236 individuals accessing IDVA services • 94.9% female • Mean 35 (SD 12.0) years old • 80.9% White British or Irish	Advocacy (IDVA): Hospital based. The key components of the role included: providing immediate support and advice, risk assessment and safety planning; referral into external services; partnership-work with hospital departments and community agencies; and training hospital staff about DVA. The most common types of support accessed were safety planning, health and wellbeing, police and housing Duration: Median case length was 1.7 months Comparator: Advocacy (IDVA): Community- based. Key components as for hospital IDVA, with the exception of training hospital staff and partnership work with hospital departments. The most common types of support accessed were safety planning, health and wellbeing, police and MARAC Duration: Median case length was 2.4 months	DVA	Cessation of abuse at time 2
Howarth 2009 [59, 61]	Grey lit	Uncontrolled pre-post	VSC	2567 individuals who explicitly engaged with services and were deemed to be high risk according to the 20-point CAADA Risk indicator Checklist and professional judgement of the assessing IDVA • All female • Average 33 years old (range 15–83) • 67% White British or Irish	Advocacy (IDVA): Seven IDVA services which varied in location (urban vs rural), size, context (dedicated IDVA vs wider community-based services), years of operation, and BME specialisation. community outreach and refuge. Most common support interventions included safety planning, support in relation to child contact, support with housing issues, and support in relation to a criminal court case services Duration: Most individuals engaged with services for at least 4 months, and 87% accessed multiple services Comparator: N/A	DVA	Cessation of all types of abuse
Howarth 2016 [60]	Peer review	Uncontrolled pre-post	Unclear	2427 individuals accessing services who had experienced recent abuse and were assessed as being at high risk for further serious abuse, defined as scooring 10 or more on a 20-item risk factor on checklist, or deemed to be at a high risk by the accessing IDVA • All female • Average 33.4 years old (SD 10.4 years) • 73% White British or Irish	Advocacy (IDVA): Seven IDVA services IDVAs indicated the areas of support received by women from a list containing 18 options that was developed through consultation with IDVA services. Areas of support include safety planning, housing and accommodation, child-related issues, access to justice systems, health and wellbeing and welfare and immigration Duration: median case length case was 99 days (1–727) Comparator: N/A	DVA	Cessation of all types of abuse
Safelives (2013) [62]	Grey lit	Uncontrolled pre-post	VCS	3242 matched cases from the SafeLives’ Adult Insights dataset • 93% female; 4% male; 1% transgender • 36% 21–30 years old • 96% heterosexual; 2% LGB • 77% White British or Irish; 21% B&ME	Advocacy (IDVA): 27 different IDVA services across England and Wales. The most common types of intervention and support included safety planning (93%), health and wellbeing advice and support (78%), MARAC (54%), and liaison/support with police (51%). The average number of interventions per client was 4.2, with 37% having 4–5 interventions Duration: Average case length was 2.2 months, with 35% having more than 10 contacts Comparator: N/A	DVA	No abuse experienced in past month / since intake
Safelives (2014) [63]	Grey lit	Uncontrolled pre-post	VCS	5549 individual and 4321 matched cases from the SafeLives’ Adult Insights dataset • 93% female; 3% male • 37% 21–30 years old • 95% heterosexual; 2% LGB • 80% White British or Irish; 19% B&ME	Advocacy (IDVA): 27 different IDVA services across England and Wales. The most common types of intervention and support included safety planning (95%), MARAC (57%) and health and wellbeing advice and support (79%). The average number of interventions per client was 4.4, with the largest majority (39%) having 4–5 interventions Duration: Average case length was 2.1 months, with the largest majority (38%) having more than 10 contacts Comparator: N/A	DVA	No abuse experienced in past month / since intake
Safelives (2017) [66]	Grey lit	Uncontrolled pre-post	VCS	4555 individual and 4026 matched cases from the SafeLives’ Adult Insights dataset • 95% female; 5% male • 35% 21–30 years old • 95% heterosexual; 2% LGB • 89% White British or Irish; 11% B&ME	Advocacy (IDVA): 43 different IDVA services across England and Wales. The most common types of intervention and support included safety planning (92%), health and wellbeing (74%), and MARAC (69%). The average number of interventions per client was 4.7, with the largest majority (37%) accessing more than 5 areas of support Duration: Average case length was 2.3 months, with most (52%) having more than 10 contacts Comparator: N/A	DVA	No abuse experienced in past month / since intake
Safelives (2019) [68]	Grey lit	Uncontrolled pre-post	VCS	3672 individual and 2311 matched cases from the SafeLives’ Adult Insights dataset • 92% female; 4% male • 37% 21–30 years old • 91% heterosexual; 3% LGB • 83% White British or Irish; 11% BME	Advocacy (IDVA): 22 different IDVA services across England and Wales. The most common types of intervention and support provided included safety (88%), mental health (51%) and housing (76%). The biggest proportion of service users (32%) received 1–5 contacts (range 1–35 +) Duration: The median case length was 12 weeks, with the largest majority (35%) being in contact for 2–3 months Comparator: N/A	DVA	No abuse experienced since intake or last review
Safelives (2021) [70]	Grey lit	Uncontrolled pre-post	VCS	2876 individual and 2360 matched cases from the SafeLives’ Adult Insights dataset • 95% female; 4% male • 34% 21–30 years old • 90% heterosexual; 2% LGB + • 86% White; 16% Black, Asian and racially minoritised people	Advocacy (IDVA): 11 different IDVA services across England and Wales. The most common types of intervention and support provided included safety (96%), housing (76%), children / parenting (65%) and mental health (61%). The average number of contacts was 14 with the largest majority (28%) having between 11–20 contacts Duration: The average (median) case length was 15 weeks, with the largest majority (33%) being in contact for 2–3 months Comparator: N/A	DVA	No abuse experienced since intake / review point
Taylor Dunn 2019 [72, 73]	Grey lit	Uncontrolled pre-post	VCS	2534 clients referred to the IDVA service in 2017 • 94.4% female; 5.2% male • 84.2% heterosexual; 0.6% homosexual or bisexual • 89.2% White; 3.2% Black; 2.1% Asian; 1.1% Mixed	Advocacy (IDVA): IDVA’s addressed the safety of victims at high risk of harm to secure their safety and the safety of their children. They served as a victim’s primary point of contact, and worked with clients from the point of crisis to assess the level of risk, discuss the range of suitable options and develop safety plans’. The service provided intensive one-to-one support within an immediate-medium term time-frame. IDVA staff worked with clients to implement Individual Support Plans and Risk Assessments within a multi-agency forum to reduce risk and encourage longer term recovery support Duration: Two thirds of cases were concluded within 5 months Comparator: N/A	DVA	Cessation in all types of abuse at exit as % of exit cases
Webster 2015 [74]	Grey lit	Uncontrolled pre-post	VCS	3031 victims of domestic violence in Kent and Medway who accessed the service	Advocacy (IDVA): The service supports the work of MARACs and the four SDVCs, with the aim of reducing the harmful effects domestic abuse has on its victims. The service employs up to 21 IDVAs at any one time, operating from 13 different districts. There is also one service manager and four administrators Duration: Not reported Comparator: N/A	DVA	Cessation of abuse in the six months from April—September 2014 based on IDVAs assessments
**Outreach**							
Safelives (2013) [64]	Grey lit	Uncontrolled pre-post	VCS	1096 individual and 941 matched cases from the SafeLives’ Adult Insights dataset • 96% female; 2% male • 32% 31–40 years old • 96% heterosexual; < 1% LGB • 88% White British or Irish; 11% B&ME	Outreach: 9 different outreach services across England and Wales. The most common types of intervention and support included health and wellbeing advice and support (91%), safety planning (83%), and housing (29%). The average number of interventions per client was 2.8, with most (60%) having 2–3 areas of support Duration: The average case length was 2.7 months, and the largest majority (43%) had less than 5 contacts Comparator: N/A	DVA	No abuse experienced in past month / since intake
Safelives (2014) [65]	Grey lit	Uncontrolled pre-post	VCS	1970 individual and 1312 matched cases from the SafeLives’ Adult Insights dataset • 92% female; 3% male; < 1% transgender • 30% 31–40 years old • 96% heterosexual; 1% LGB • 84% White British or Irish; 14% B&ME	Outreach: 16 different outreach services across England and Wales. The most common types of intervention and support included safety planning (88%), health and wellbeing support and advice (79%), and housing (35%). The average number of interventions per client was 3.0, with most (54%) having 2–3 areas of support Duration: The average case length was 2.6 months, and the largest majority (47%) had less than 5 contacts Comparator: N/A	DVA	No abuse experienced in past month / since intake
Safelives (2017) [67]	Grey lit	Uncontrolled pre-post	VCS	3510 individual and 3187 matched cases from the SafeLives’ Adult Insights dataset • 94% female; 6% male • 32% 21–30 years old • 94% heterosexual; 1% LGB • 91% White British or Irish	Outreach: 28 different Outreach services across England and Wales. The most common types of intervention and support included safety planning (86%), health and wellbeing (78%), and housing (37%). The average number of interventions per client was 3.3, with the largest majority (45%) having 2–3 areas of support Duration: The average case length was 1.9 months, and the largest majority (40%) had more than 10 contacts Comparator: N/A	DVA	No abuse experienced in past month / since intake
Safelives (2019) [69]	Grey lit	Uncontrolled pre-post	VCS	1820 individual and 1086 matched cases from the SafeLives’ Adult Insights dataset • 90% female; 5.2% male • 33% 31–40 years old • 90% heterosexual; 2.2% LGB • 83% White; 13% BME	Outreach: 22 different Outreach services across England and Wales. The most common types of intervention and support provided included safety (77%), mental health (67%), and housing (76%) Duration: The average (median) case length was 13 weeks, with the largest majority (36%) having 2–3 months of support and 44% having 1–5 contacts Comparator: N/A	DVA	No abuse experienced since intake or last review
Safelives (2021) [71]	Grey lit	Uncontrolled pre-post	VCS	3211 individual and 2149 matched cases from the SafeLives’ Adult Insights dataset • 91% female; 7% male • 35% 31–40 years old • 88% heterosexual; 2.3 LGB + • 81% White; 10% Black, Asian and racially minoritised	Outreach: 11 different Outreach services across England and Wales. The most common types of intervention and support provided included safety (84%), MARAC (58%) and mental health (57%) Duration: The average (median) case length was 18 weeks, with the largest majority (26%) having 2–3 months of support and 28% having 1–5 contacts Comparator: N/A	DVA	No abuse experienced since intake or last review
**Psychological support**							
Calvert 2015 [75]	Peer review	Uncontrolled pre-post	Public	157 people with a history of child sexual abuse, and this abuse played a central role in their ongoing psychological distress, disorganized engagement patterns with services, elevated risk, and/or poor self-care • All female • Mean 34.65 years old (range 18–64) • 88% White; 4% non-White; 8% Unknown	Psychological support: Group Cognitive Analytic Therapy (CAT), using psychoanalytic and cognitive models to offer a transdiagnostic, time-limited and relational approach to facilitating therapeutic change, following a reformulation, recognition, and revision approach. Specific interventions varied over time and in response to the needs of the members and group dynamics. Duration: weekly sessions lasting 90 min for 24 weeks Comparator: N/A	CSA	Rosenberg Self Esteem Scale measured at assessment, pre-treatment (during first session) and at final session
Clarke 1994 [76]	Peer review	Uncontrolled pre-post	Public	7 survivors of childhood sexual abuse committed by a male perpetrator • All female • Mean 27 years old (range 19–48 years)	Psychological support: Cognitive analytic therapy (CAT), which involves a collaborative reformulation of clients’ difficulties, manifested in neurotic or maladaptive patterns of behaviour. Uses psychodynamic, cognitive and behavioural techniques, with strategies such as cognitive restructuring, reprocessing of nightmares, assertiveness training, and most crucially, interpretation of transference and counter-transference Durations: 16 weeks Comparator: N/A	CSA	Rosenberg Self-Esteem Scale, measured at baseline, 16 weeks and 3 month follow up
Clarke 2000 [77]	Peer review	Uncontrolled pre-post	Public	4 survivors of childhood sexual abuse • All male • Aged 22–53 years	Psychological support: Cognitive Analytic Therapy (CAT) in an outpatient setting Duration: 16 sessions Comparator: N/A	CSA	Rosenberg Self-Esteem Scale, measured at baseline and 16 weeks
Ellis 2012 [78]	Peer review	Uncontrolled pre-post	VCS	59 adult survivors of historic child sexual abuse • All female	Psychological support: Group therapy that draws on cognitive behavioural therapy, strengths based theory, systemic therapies, brief therapy and positivism, involving learning how to cope with flashbacks, memories and feelings of anger and grief. Methods included journal work, recovery writing and art and sensory therapy Duration: 2 h sessions over 8 weeks Comparator: N/A	CSA	Rosenberg Self-Esteem Scale. Only presented graphically
Karatzias 2016 [79]	Peer review	Uncontrolled pre-post	Public	82 service users with a history of interpersonal trauma (child sexual abuse, child neglect, physical abuse, assault, domestic violence) and subsequent psychological distress (i.e., traumatic symptomatology, dissociation, self-esteem, general distress) • All female • Aged 18–65 years old	Psychological support: Trauma Recovery and Empowerment Model (TREM). A group-based therapy drawing on cognitive-behavioural and skills training techniques, including self-comfort, self-monitoring, establishing boundaries, reframing experiences, communication and decision-making skills and regulating feelings. Groups consisted of a up to 10 participants Duration:18 90-min sessions Comparator: N/A	DVA, SVA, CSA	Rosenberg Self-Esteem Scale reported at pre-, mid-, post-intervention and follow-up
Smith 2015 [80]	Peer review	Non-randomised comparative / pre-post	VCS	158 mothers and children who have experienced domestic abuse A comparison group was also recruited, and these data were collected by a women’s refuge service which supported mothers and children who experienced domestic abuse	Psychological support / parenting programme: Domestic Abuse Recovering Together (DART) focuses on rebuilding the mother–child relationship after the abuse has ended, reducing difficulties experienced by children, increasing self-esteem and mothers’ confidence in their parenting skills. Some are joint sessions, and some are separate Duration: Weekly for 10 weeks, with sessions lasting 2–2.5 h Comparator: A small comparison group was drawn from a play therapy group. No further details	CSA	Rosenberg Self Esteem Scale. Measured before the programme, end of programme and at 6 months follow-up
**Perpetrator programmes**							
Bowen 2003 [81–85]	Peer review & grey lit	Uncontrolled pre-post	Public	120 male domestic violence offenders • All male • Average (SD) age: 34.95 (9.04) years White British 85%; Asian 9%; Afro-Caribbean 3%; Turkish 3%	Perpetrator programme: The programme is a pro-feminist psychoeducational treatment programme with 5 modules focusing on the offenders' own definitions of DV and the behaviour targeted by the programme; male socialisation and adoption of patriarchal attitudes being associated with DV; the impact of DV on partners and children, and empathy for victims; sexual respect in intimate relationships, and accountability and communication within relationships Duration: 24 2.5-h group sessions once or twice a week, and five 2.5 h follow up sessions once per month Comparator: N/A	DVA	Balanced Inventory of Desirable Reporting
Gilchrist 2021 [86, 87]	Peer review	Randomised controlled trial	Mixed	104 men who had perpetrated abusive or violent behaviour towards a current or ex-partner in the last 12 months • All male • Mean (SD) age: 42.1 (10.1) years • White 75.4%; Black 10.7%; Asian 9.7%; other 3.9% 27 current or ex-partners of men participating in the trial • All female • Mean (SD) age: 41.8 (12.1) years • White 59.3%; Black 11.1%; Asian 18.5%; Other 11.1%	Perpetrator programme: ADVANCE is a manualised evidence-informed tailored intervention developed to target IPA perpetration by men attending substance use treatment. It focuses on developing healthy relationships, enhances reflective motivation, and challenging sexist and patriarchal beliefs. It uses education, self-regulation, goal setting with a cognitive and dialectical behavioural and motivational enhancement approaches Duration: 2–4 individual sessions followed by 12 weekly group sessions Comparator: Usual care. Male participants in both treatment arms received substance use treatment as usual, including group work, individual sessions, mutual aid and opiate substitution treatment Duration: Weekly groups and fortnightly individual sessions, duration not reported	DVA	Balanced Inventory of Desirable Reporting
Lindsay 2011 [88]	Peer review	Uncontrolled pre-post	Unclear	15 men with intellectual disabilities who had offended against adult women • All men • Mean age: 32.7 years	Perpetrator programme: Group sex offender treatment programme with modules on disclosure, pathways into offending, personal issues related to cognitive distortions, childhood abuse, victim awareness, interpersonal style, use of pornography and relapse prevention techniques promoting adaptive attachment, engagement with society and a relapse prevention plan Duration: Weekly 2-h sessions for 26 months Comparator: N/A	SVA	Questionnaire on Attitudes Consistent with Sexual Offending (QACSO)
Murphy 2007 [89]	Peer review	Uncontrolled pre-post	Public	10 men with sexually abusive behaviour and intellectual disability • All men • Mean (SD) age: 38.8 (14.6) years	Perpetrator programme: Group cognitive behavioural therapy for sexually abusive behaviour, including sessions on body part names, social rules for dressing/undressing and touching, social and sexual relationships, what is legal, illegal and risky, coping with feelings, consequences of their behaviour, descriptions of their illegal sexual behaviour, how hard it is to talk about illegal sexual behaviours and how men cope with this (including denial, minimization, victim blaming), experiences of being victims themselves, how others feel when they are victims, how their own victims felt, causes of their own sexual behaviour, understanding offence cycles, understanding choice, consent and age of consent, and relapse prevention Duration: Weekly 2-h sessions for 1 year Comparator: N/A	SVA	Questionnaire on Attitudes Consistent with Sexual Offending (QACSO)
Ormston 2016 [90]	Grey lit	Uncontrolled pre-post	VCS	941 men who have been convicted of offences involving domestic abuse and were assessed as moderate or high risk of future domestic abuse • All male • 16–18 years 1%; 19–24 years 17%; 25–34 years 44%; 35–44 years 23%; 45–54 years 12%; 55 + years 3% • White 98%; African, Caribbean or Black 0.5%; Asian, Asian Scottish or Asian British 0.4%; Mixed or multiple ethnic groups 0.3%; Other 1% 598 female (ex)partners	Perpetrator programme: The Men’s Programme is a manualised programme aimed at changing men’s behaviour, including both one-to-one and group work Duration: At least 2 years: 14 one-to-one preparation and motivation sessions, 26 weekly 3-h group sessions, and further one-to-one post-group sessions Comparator: N/A	DVA	Balanced Inventory of Desirable Reporting
Rose 2012 [91]	Peer review	Uncontrolled pre-post	Public	12 sexual offenders with severe intellectual disability in a community setting • All male • Mean (range) age 39.5 (20–65) years	Perpetrator programme: Group programme partly based on an existing sex education and relationships programme. Included sessions on motional recognition in themselves and others; examining their life stories and analysing motivation to offend; describing their offences in detail and introducing the offence cycle; anger management; excuses, cognitive distortions and alternatives to offending; victim empathy; relapse prevention. Behaviours and cognitions are challenged, and participants are given opportunities to reflect Duration: Weekly 2-h sessions for 40 weeks Comparator: N/A	SVA	Questionnaire on Attitudes Consistent with Sexual Offending (QACSO)

*Note CSA* child sexual abuse, *DVA* domestic violence and abuse, *DV* domestic violence, *SD* standard deviation, *SVA* sexual violence and abuse, *VCS* voluntary and community sector

#### Interventions

##### Advocacy/IDVAs

Eleven studies reported on IDVA services [57, 59–61, 63, 64, 67, 69, 71, 73, 75]. Eight of the studies provided data from multiple IDVA services, representing a total of 158 IDVA services between them. Five of the eight studies were SafeLives Insights reports. All but two of the studies were found in the grey literature search, and the majority (n = 9) were located in the VCS. One was a mix of sectors, with one hospital-based IDVA (public sector) and one community-based IDVA (VCS), and for one the sector was unclear. Most studies used an uncontrolled pre-post design (n = 10), while one used a non-randomised comparative study design. The eight studies that reported on multiple IDVA services did not describe the individual services in detail, however they did report the usage of various types of support interventions. The most commonly accessed support intervention as part of the IDVA service for all reports was safety planning. Other forms of support commonly accessed included housing, mental health, child-related issues, and multi-agency risk assessment conferences (MARACs). Of the three studies that evaluated a single IDVA service, one compared a hospital based IDVA to a community-based IDVA, one described an IDVA service that supported the work of MARACs and four specialist domestic violence courts (SDVCs), and one described an IDVA service that offered intensive one-to-one support in a medium-term timeframe, that focused on safety planning and risk assessments.

##### Outreach

Five studies provided data for a total of 86 outreach interventions [65, 66, 68, 70, 72]. All five studies were SafeLives Insights reports. All were found in the grey literature search, all used uncontrolled pre-post designs, and all services were located in the VCS. Because of the nature of the SafeLives Insights measurement service and the datasets it produces, details of the included outreach services are not provided. However, for each publication, the types of intervention and support accessed by people using the outreach service are reported. For all but one publication, the most commonly accessed type of support was safety planning, while for one publication health and wellbeing advice and support was the most commonly accessed support type. The average duration of outreach support ranged from 1.9–4.5 months.

##### Psychological support

Six studies reported on psychological support interventions [76–81]. All of these were peer reviewed, and five used uncontrolled pre-post designs. One study included a comparator group, but data on self-esteem were not collected for this group, thus only data from the intervention arm were included. Two of the six interventions were in the VCS, while the rest were based in the public sector. Three of the studies used Cognitive Analytic Therapy, which uses a mix of psychodynamic, cognitive and behavioural techniques to aid reprocessing, assertiveness, and transference. One study described a Trauma, Recovery and Empowerment Model, which is a group based cognitive-behavioural therapy. One study reported a parenting programme called Domestic Abuse Recovering Together, which focuses on rebuilding mother–child relationships and increasing confidence and self-esteem. One paper reported a group therapy which involved journal work, recovery writing and art therapy. The duration of the support interventions ranged from eight to 24 weeks.

All but one study focused exclusively on adults who had experienced child sexual abuse. One included people with a history of interpersonal trauma, including child sexual abuse, neglect, physical abuse, domestic violence or assault. Five of the six studies comprised of women only, while one study only included men.

##### Perpetrator programmes

Six studies evaluated perpetrator programmes, three reporting the Questionnaire on Attitudes Consistent with Sexual Offending (QASCO) [89, 90, 92], and three reporting the Balanced Inventory of Desirable Reporting (BIDR) [82, 88, 91]. Of the three reporting the BIDR, all were aimed at men who had previously perpetrated domestic abuse. Two of the studies were peer reviewed, and one was found in the grey literature. One used a randomised controlled trial design, whilst the other two used uncontrolled pre-post designs. One was based in the VCS, one in the public sector, and one was mixed. The programme described by Gilchrist (2021) was a behaviour change intervention developed using the Behaviour Change Wheel and the COM-B model of behavioural interventions, while the programme used by Bowen (2003) used a psychoeducational approach, and the programme described by Ormston (2016) utilised a systems approach to change men’s behaviour which also works with women and children. The three studies reporting the QASCO all recruited men with intellectual disability who had sexually assaulted women. All three studies were peer-reviewed, used a pre-post design (one used a comparative study design but the second arm was excluded as the population were men who had perpetrated against children, which is outside of the scope of this review), and two were based in the public sector, while for the third study the setting was unclear. All three programmes used group work and focused on understanding their behaviour, addressing cognitive distortions, and prevention relapse.

## Effects of the interventions and services

### Advocacy/IDVAs

**Fig. 2:**
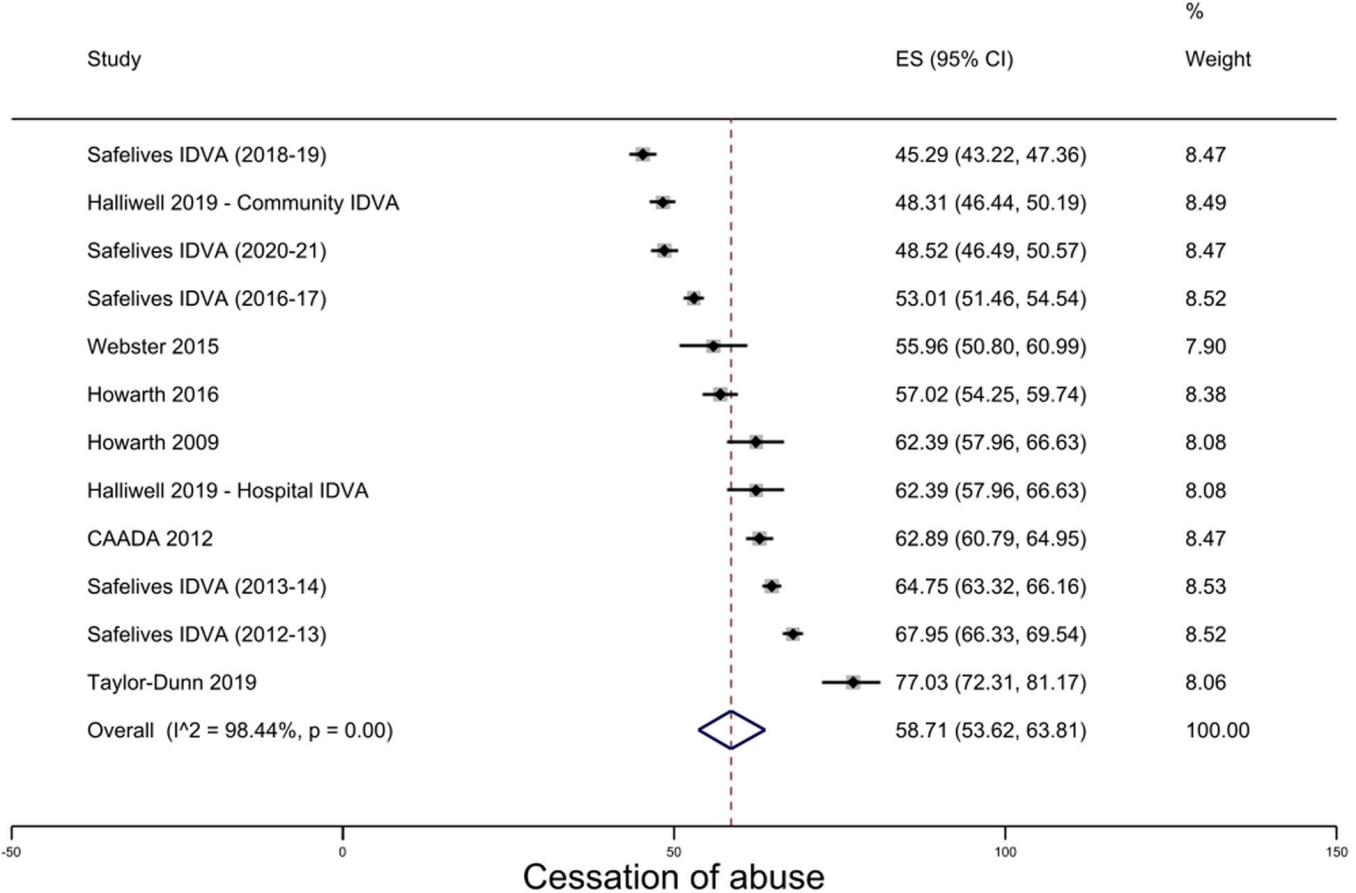
Cessation of abuse at case closure / end of intervention for advocacy/IDVA services

Twelve arms from eleven studies were included in the meta-analysis (Fig. 2). All showed an increase in participants reporting cessation of abuse from pre- to post-intervention (i.e., at case closure). The overall pooled prevalence of cessation in abuse was 58.7% (95% CI 53.6–63.8). The IDVA service reported by Taylor-Dunn and Erol (2019) showed the greatest increase in participants reporting cessation of abuse (77.0%, 95% CI 72.3–81.2), while the dataset collating data from 22 IDVA services produced by SafeLives in 2019 showed the lowest increase (45.3%, 95% CI 43.2–47.4).

Heterogeneity levels were very high (I^2^ = 98.4%; Cochran’s Q: χ^2^(10) = 703.7, p < 0.01), however planned subgroup analyses could not be undertaken because for each analysis, either one subgroup had less than three contributing studies (e.g., study design, sector, type of violence), or studies did not report enough information (e.g., relationship to the perpetrator, type of provider).

### Outreach

**Fig. 3:**
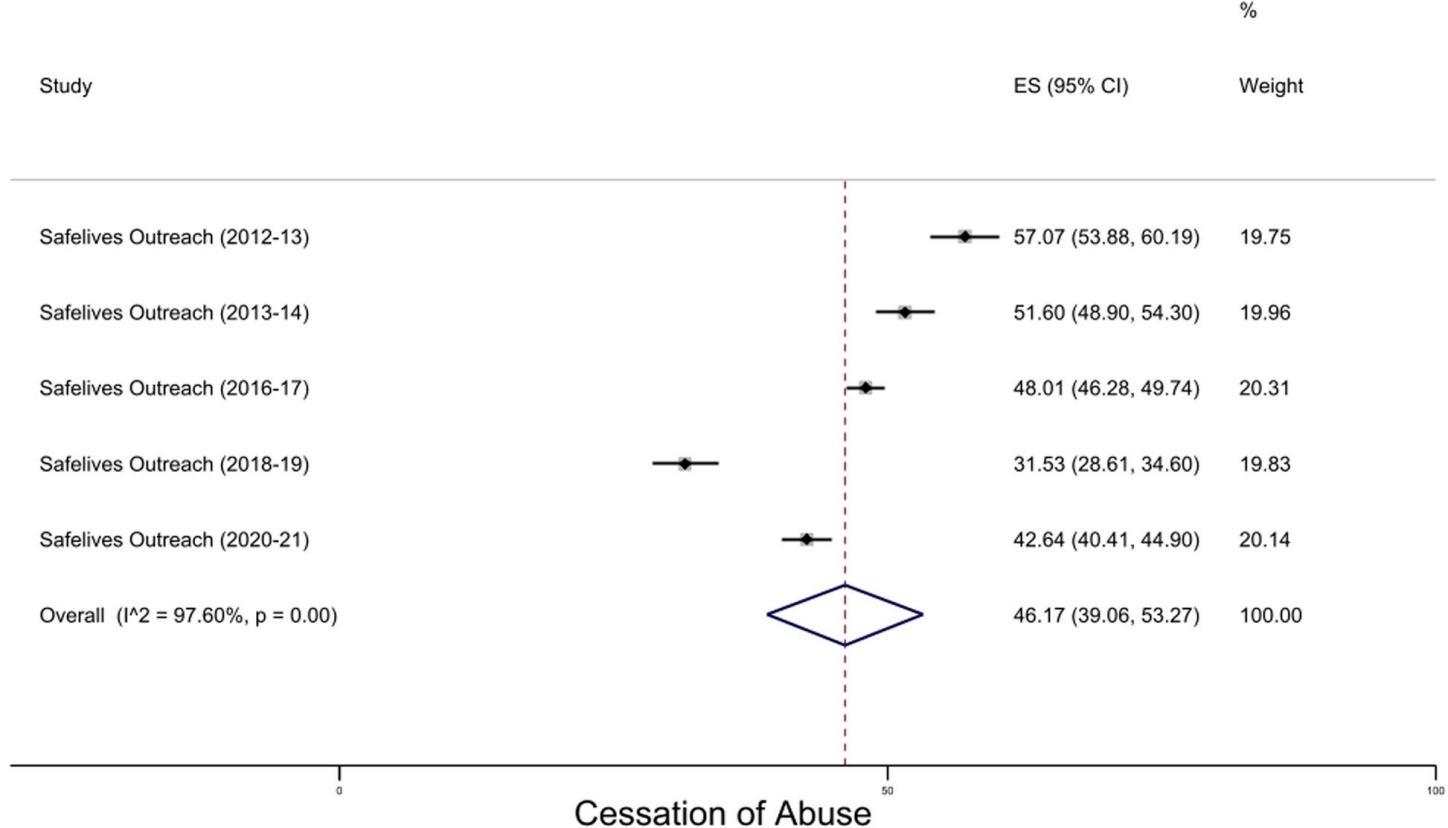
Cessation of abuse at case closure / end of intervention for outreach services

Five studies were included in the meta-analysis (Fig. 3). All showed an increase in cessation of abuse from pre- to post-intervention. The overall pooled prevalence of abuse cessation was 46.2% (95% CI 39.0–53.2). Individual prevalence ranged from 31.5% to 57.1%. As with advocacy interventions and services, there was very high heterogeneity (I^2^ = 97.6%; Cochran’s Q: χ^2^(10) = 166.5, p < 0.01). Planned subgroup analysis to explore the potential causes of this could not be carried out because all studies fell into the same category (i.e., study design, sector, source of literature, type of violence, type of provider, relationship to perpetrator).

### Psychological support

The Rosenberg Self-Esteem Scale was reported by six studies, however one only reported results graphically, therefore mean scores could not be extracted. None of the remaining studies reported enough data for robust meta-analysis, therefore synthesis was conducted using vote counting based on the direction of effect. This showed that all studies showed a positive impact of psychological support interventions on the outcome (see Table 2 for the effect direction table).

**Table 2:** Effect direction table summarizing direction of impacts from studies of psychological support interventions

Study ID	Study Design	Risk of Bias	Sample size	Intervention duration	RSES
Calvert 2015	Pre-post	Serious	157	24 weeks	▲
Clarke 1994	Pre-post	Serious	7	16 weeks	▲
Clarke 2000	Pre-post	Serious	4	16 sessions	▲
Ellis 2012	Pre-post	Serious	59	8 weeks	▲^b^
Karatzias 2016	Pre-post	Serious	82	18 sessions	▲
Smith 2015	NRC^a^	Serious	158	10 weeks	▲

*NRC* non-randomised comparative study, *RCT* randomised controlled trial

Effect direction: upward arrow ▲ = positive impact, downward arrow ▼ = negative impact, sideways arrow ◄► = no change/mixed effects/conflicting findings

Sample size: Final sample size (individuals) in intervention group – large arrow ▲ > 300; medium arrow ▲ 50–300; small arrow ▲ < 50

^a^Study design is non-randomised comparative however this outcome was not measured in the comparison group

^b^Results are reported graphically

### Perpetrator programmes

Meta-analysis was not possible for either the BIDR or the QASCO outcomes, due to either insufficient reporting (i.e., standard deviations not being reported), or discrepancies between studies in terms of whether the total score or subscale scores were reported. Therefore, both perpetrator programme outcomes were synthesised using the vote counting methods, and results are presented in Table 3. All three perpetrator programmes reporting the QASCO showed positive impacts on the outcome, although all had small sample sizes. For the BIDR, Bowen (2003) showed a slight increase in impression management, and a significant increase in terms of the self-deception subscale. Gilchrist et al., (2021) found no change from baseline to end of treatment, whilst Ormston et al., (2016) found a slight increase in self-deception but no change in impression management.

**Table 3:** Effect direction table summarizing direction of impacts from studies of perpetrator programmes

Study ID	Study Design	Risk of Bias	Sample size	Intervention duration	QASCO	BIDR
Lindsay 2011	Pre-post	Serious	15	36 months	▲	
Murphy 2007	Pre-post	Serious	8	1 year	▲	
Rose 2012	Pre-post	Serious	12	40 weeks	▲	
Bowen 2003	Pre-post	Issues detected	27	~ 8 months		▼
Gilchrist 2021	RCT	High	54	16 weeks		◄►
Ormston 2016	Pre-post	Issues detected	130	26 weeks		◄►

*BIDR* Balanced Inventory of Desirable Reporting, *RCT* randomised controlled trial, *QASCO* Questionnaire on Attitudes Consistent with Sexual Offending

Effect direction: upward arrow ▲ = positive impact, downward arrow ▼ = negative impact, sideways arrow ◄► = no change/mixed effects/conflicting findings

Sample size: Final sample size (individuals) in intervention group – large arrow ▲ > 300; medium arrow ▲ 50–300; small arrow ▲ < 50

#### Sensitivity analyses

We were unable to perform sensitivity analysis by removal of high risk of bias studies as all studies were assessed as having high risk of bias. Sensitivity analysis removing one study at a time was conducted for meta-analysed outcomes (Additional file 5). For both outcomes, removing each study did not substantially change the estimates.

#### Quality and certainty assessments

**Fig. 4:**
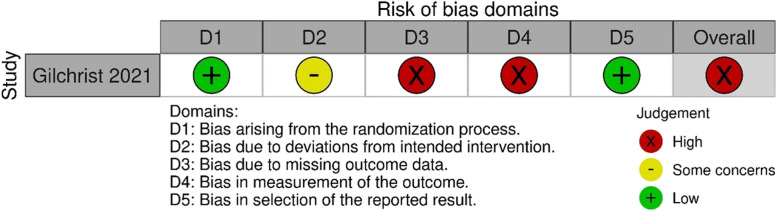
Risk of bias using the RoB2 tool

One randomised controlled trial [88] was assessed using the RoB2 tool. This study was assessed as having a high risk of bias, due to concerns regarding missing data and measurement of the outcome (Fig. 4; Table A1).

**Fig. 5:**
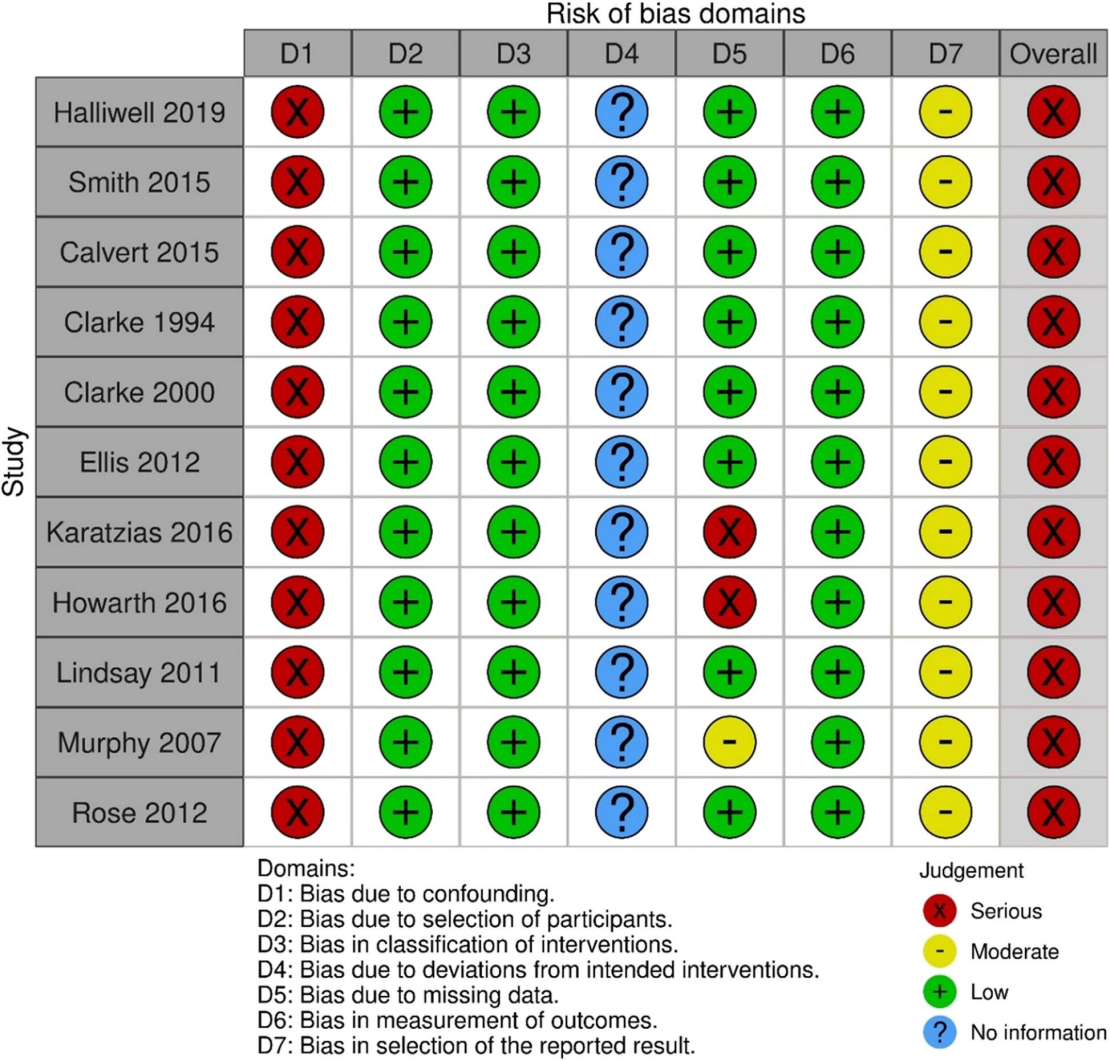
Risk of bias using the ROBINS-I and the adapted ROBINS-I tools

Two non-randomised comparative trials [59, 81] were assessed using the ROBINS-I tool. Both studies were determined to have a serious risk of bias, primarily due to concerns regarding confounding variables, missing data, measurement of the outcome due to lack of blinding, and selection of the reported result as neither study had pre-registered protocols available (Fig. 5; Table A1).

Nine uncontrolled before and after studies [61, 76–80, 89, 92, 93] were assessed using an adapted version of the ROBINS-I tool. All were judged as having a serious risk of bias. This was again primarily related to issues with potential confounding, some issues with missing data and some concerns regarding a lack of protocol meaning that there may be potential for selected reporting (Fig. 5; Table A1).

Seventeen studies [57, 60, 63–73, 75, 82, 91, 94] found in the grey literature were assessed using the AACODS checklist. While this tool does not provide an overall risk of bias rating, it does allow for the identification of key quality issues, which included concerns regarding a lack of detailed reference lists or sources for some of the publications, lack of transparency regarding limits of the research, and some concerns regarding significance (Table A2).

Evidence certainty was assessed using GRADE. For studies that were not meta-analysed, GRADE assessments were conducted following published guidance [95]. Taking into account the above risk of bias ratings, inconsistency, indirection, impression and publication bias, the certainty of evidence rating was very low for both cessation of abuse and for desirable responding, low for attitudes towards sexual offending, and moderate for self-esteem. Full details of the assessments can be found in Additional file 4 (Table A3).

## Discussion

This review is the first to assess the effectiveness of multiple types of support services and interventions for people who have experienced DSVA in the UK, using a comprehensive search strategy encompassing both the peer-reviewed and grey literature, and drawing upon a stakeholder advisory group to guide the development and progress of the review. This review aimed to determine the effectiveness of support interventions and services at improving the safety and wellbeing of those affected by DSVA.

### Overview of findings

The review found that both advocacy/IDVA services and outreach services had a positive effect in terms of the proportion of service users reporting that the abuse had ceased by case closure. These findings broadly concur with previous systematic reviews based on evidence primarily from the USA, which have concluded that there is weak support for advocacy in terms of cessation or reduction of some types of abuse, improved quality of life and improved mental health, but that further research and evaluation is necessary [36, 96].

The results for psychological support services similarly suggested a positive effect on self-esteem, with all studies showing a positive direction of effect. This is also broadly reflective of the international evidence, with one meta-analysis of three studies showing non-significant improvements in self-esteem following various forms of psychological support interventions, including cognitive trauma therapy, an empowerment programme, and stress management [97], and another review showing improvements in self-esteem and other wellbeing related outcomes following counselling interventions [98].

In terms of perpetrator programmes, results were more mixed. For the three studies reporting attitudes consistent with sexual offending, all studies showed effects consistent with a positive impact, although this evidence is limited only to sexual offenders with intellectual disabilities and cannot be generalised to other perpetrators of DSVA. Attitudes towards violence has been listed as one of the key factors underpinning prevention of violence perpetration, therefore this does suggest that there may be benefits in reducing violence perpetration. Results for the desirable reporting outcome showed either no effect or negative effects (i.e., increased levels of desirable reporting after the programme). It should be noted that while the BIDR was reported in the three perpetrator programmes as an outcome, with pre- and post-intervention values reported, it’s intended use is to assess socially desirable reporting so that other self-reported scales of interest can be adjusted for, rather than being an outcome in and of itself. Thus, it would not necessarily be expected that a perpetrator programme would result in changes to social desirability, therefore these findings are not surprising.

### Discordance between review findings and stakeholder views

A major strength of this review was the involvement of the stakeholder advisory group, whose insight in terms of providing context, developing the scope and advising on analysis approaches was invaluable. The stakeholder consultation process also provided some unexpected challenges and incidental findings, such as when there were discrepancies between the evidence and stakeholder views. For instance, stakeholders were disappointed that some of the outcomes that they considered most important and relevant to service users and deliverers were not reflected in the findings of the review. As an example, some of the outcomes that were valued by the stakeholders could not be included in the review due to either lack of evidence, too much variation in how they were specifically operationalised, or because the way in which they were operationalised did not meet the eligibility criteria of the review (i.e., they were not measured at more than one time-point). For instance, stakeholders considered women’s self-reported perception of their safety a key outcome of perpetrator programmes, however this could not be included in the review because it was often assessed retrospectively at the end of the intervention only or, when assessed at two time-points, there was too much divergence in how it was measured. On the other hand, the stakeholders considered *cessation of abuse* as an outcome of support services unrealistic. It was clear that for stakeholders the priority was to make those who have experienced DSVA safer, but that striving for perfection (i.e., complete cessation of all abuse, rather than a reduction in the frequency, severity and/or duration of DSVA), was unfeasible, and would likely understate the impact of the service. While cessation of abuse may be the ultimate long-term goal, other short and medium-term goals that focus on enhancing safety over time are more achievable.

### Challenges

A challenge in terms of both evidence synthesis in this field and for those commissioning and delivering DSVA services is the large variation and inconsistency in outcomes being measured to assess service and intervention effectiveness. This is largely driven by funding bodies and the fragile and fragmented funding landscape of DSVA services in the UK. Often various bodies are involved in the funding of a service, each with their own agenda and stipulations as to what service deliverers need to measure to assess effectiveness. This can lead to a single service being required to capture multiple forms of data and outcomes to fulfil different funders’ requirements, and these data and outcomes differing between services. Additionally, these required outcomes may be at odds with the service deliverers’ own concept of effectiveness, which may result in services choosing to collect further outcomes, where resources allow. A third contributing factor to the variation in outcomes measured is that some services, where funding allows, commission independent service evaluations, which often require additional outcomes to be measured. Thus, the outcomes measured may reflect differing agendas or understandings of what is an important measure of effectiveness.

The above has two consequences relevant to this review. First, the outcomes reported in the included studies may be reflective of what funders require services to report, rather than what service deliverers view as most important or relevant to those they are supporting, or what is most meaningful in the lives of victim-survivors and perpetrators. This may explain the discrepancies noted above in terms of stakeholder outcome preferences compared to those identified in the literature. The second issue is that by including these outcomes in the review, we run the risk of reinforcing that this is how effectiveness should be measured in this field. Therefore, it is important to acknowledge that while the outcomes utilised in this review represent the most consistently used and therefore amenable to synthesis through meta-analysis, they should not necessarily continue to be used if they are not the outcomes that are valued most by service providers and people with lived experience. Instead, focus should be on building up the evidence base for those outcomes that are most valued, identifying them through co-production with survivors and service providers, in a consistent way (i.e., using consistent outcome measurement tools), which will allow for more meaningful syntheses in the future. This may mean increased consistency in funders’ requirements and more sustainable funding to facilitate this data collection.

A further challenge to synthesis through meta-analysis is the inconsistency in how robustly outcome data are reported. This challenge is illustrated in this review. The methods used to identify outcomes should have ensured that meta-analysis was possible for all outcomes. However, whilst meeting the criteria for the review (i.e., three or more studies reporting the same outcome and using the same outcome measurement tool), three could not be meta-analysed due to insufficient or inconsistent reporting (i.e., not reporting standard deviations, only reporting results graphically, use of subscale scores versus total scores). Thus, inconsistency is an issue both in terms of the outcomes used and how they are reported.

To address this in the future, and allow for subsequent meta-analysis that can be more inclusive, we recommend improving reporting practices by following best practice guidance. Reporting guidelines exist for a range of study types, including randomised trials (CONSORT 2010 [99]), observational studies (The Strengthening the Reporting of Observational Studies in Epidemiology (STROBE) Statement [100]), and quality improvement studies (SQUIRE 2.0 – Standards for QUality Improvement Reporting Excellence [101]). While there is no reporting guidance specifically for service evaluations, some of the guidance for other designs do apply. In particular, it is important that if the aim is to demonstrate improvement, change, or impact, outcomes need to be assessed at more than one time point. To facilitate meta-analysis, authors should report mean values with a measure of variation (i.e., the standard deviation), and clearly report the number of individuals who completed the outcome measure at each time point. It is also important to avoid only presenting data graphically. Better reporting, together with more consistency in outcome measures used, will enable larger, and therefore more powerful synthesis in the future.

### Strengths and limitations

A major strength of this review is the inclusion of a comprehensive grey literature search strategy. This allowed for identification of reports and evaluations carried out by specialist support services that are not peer-reviewed or identifiable via traditional literature databases, thus reducing publication bias and allowing identification of a wider range of reports. As already noted, the continued involvement of stakeholders was another strength, as this group provided essential guidance on the review as it developed and ensured that the review process was sensitive to the context and the various political, financial and ethical issues and considerations. A limitation of our approach to stakeholder engagement was that we did not explicitly invite input from a lived experience perspective. Whilst many service providers in the domestic abuse sector also have lived experience of DSVA, the input we sought was from a service provider perspective. The insights we gained may have been further strengthened had we also gathered input from a lived experience perspective.

There are several further limitations to the evidence produced by this review. First, all of the peer-reviewed literature had a high risk of bias, primarily due to confounding factors and a lack of information provided, such as a study protocol. The grey literature should be interpreted with the understanding that it has not undergone a peer-review process. Additionally, quality appraisal of grey literature studies highlighted concerns about authority, accuracy and significance. Second, because of the inconsistency surrounding outcome reporting, three of the included outcomes could not be meta-analysed. Vote counting was used instead, based on the available data. This method is only able to determine whether there is any evidence of an effect, rather than what the average effect is, limiting the conclusions that can be drawn. Third, much of the evidence, particularly for advocacy/IDVA services and outreach services, is based on publications from one service provider (SafeLives), but there is insufficient information regarding the structure and provision of each service represented by the data. It is possible that a service may self-define as advocacy, but a similar service may define itself as an outreach service. Similarly, the specific forms of support offered by advocacy/IDVA and outreach services appear similar (e.g., according to the SafeLives Insights reports, both frequently report safety planning and housing as common forms of support offered and accessed). Therefore, there may be overlap between the categories of services, but because information on how they self-define and descriptions of each contributing service are not reported, the extent of this cannot be determined. A final limitation, as explained above, this review only speaks to evidence for the outcomes that were most commonly measured, which is not necessarily synonymous with being the most relevant or useful outcomes. The danger of this is perpetuating a flawed system where services are evaluated on outcomes that are not necessarily consistent with their aims or ethos. To avoid this, we are clear that this review provides evidence for the effectiveness of support interventions based on the available data, but that work needs to be done to ensure that the most relevant and useful outcomes are measured consistently, to aid services in evidencing their effectiveness and to enable more meaningful syntheses of the evidence in the future.

### Implications and future directions

This review highlights the value of UK-based advocacy and outreach interventions for reducing DSVA revictimisation, of psychological support for improving self-esteem, and of perpetrator programmes for improving attitudes to sexual offending. However, the lack of high-quality evidence means that there is some uncertainty regarding the effect estimates. There is a need for high quality research that incorporates randomisation between interventions, where appropriate and ethical. Research practices such as publishing of study protocols, following reporting guidelines and, for research where randomisation is not feasible, considering and accounting for potential confounding factors, would greatly improve the quality and robustness of research in this field.

Another way to improve the robustness of the evidence base would be greater consistency in outcomes being measured to assess effectiveness and greater consensus between researchers, service providers, and funders. Core-outcome sets have been developed through co-production with survivors, practitioners, commissioners, policymakers and researchers, in related areas such as child and family-focused interventions for child and domestic abuse [102]. Developing a core-outcome set specific to adult DSVA that reflects the short and medium-term goals that both service providers and survivors value, building on existing efforts that have been made in this area [103], and underpinning a theory of change towards ending violence, will facilitate cohesion and the development of a robust evidence-base.

It is important to acknowledge that the theory underlying perpetrator programmes in particular is evolving, with recent evidence from the US indicating a shift from traditional approaches, such as psychoeducation and CBT, towards trauma-informed approaches that focus more on the consequences of trauma that may lead to violence perpetration (e.g., [104]). Of the six UK-based perpetrator programmes identified in the current review, traumatic experiences and the potential benefits of using a trauma-informed approach are briefly mentioned in two. However, it is not clear if either programme did go on to incorporate these practices into the development of the interventions. Recent literature suggests that in the UK, trauma-informed perpetrator programmes are being developed and used [105, 106], however this work is still in its infancy. Future work in this area should therefore consider the evidence for more trauma-informed perpetrator programmes and look to assess the effectiveness of such programmes in the UK.

Finally, whilst this review focused on quantitative data to address the review question, there is a wealth of qualitative data that addresses the impact of support interventions on people who have experienced DSVA. Therefore, synthesis of this qualitative evidence would be valuable to complement the current review and provide a more holistic and representative overview of the evidence contributing to this field.

## Supplementary Information

Additional file 1. Checklists (PRISMA and SWiM). Contains the Preferred Reporting Items for Systematic Reviews and Meta-Analyses (PRISMA) and Synthesis Without Meta-analysis (SWiM) reporting checklists.

Additional file 2. Example search strategy. Contains an example search strategy for one of the peer reviewed literature databases (Medline) and one of the grey literature databases (Social Care Online).

Additional file 3. Risk of bias assessments. Contains tables A1 and A2, detailing the risk of bias assessments for randomised controlled trials, non-randomised comparative trials and uncontrolled before and after studies (Table A1), and for the grey literature (Table A2).

Additional file 4. GRADE Certainty Assessment. Contains Table A3, which details the assessments of certainty for each of the outcomes using the GRADE framework.

Additional file 5. Sensitivity analyses (leave one out analysis). Contains Figures A1 and A2 which show the leave one out analyses for the Cessation of Abuse outcome for advocacy/IDVA interventions outcome, and the Cessation of Abuse outcome for outreach interventions outcome.

## Data Availability

The datasets used and/or analysed during the current study are available from the corresponding author on reasonable request.

## Abbreviations

ASSIA: Applied Social Sciences Index & Abstracts
BIDR: Balanced Inventory of Desirable Reporting
CI: Confidence interval
DSVA: Domestic and sexual violence and abuse
DVA: Domestic violence and abuse
EMBASE: Excerpta Medica Database
IBSS: International Bibliography of the Social Sciences
IDVA: Independent domestic violence advocate
MARAC: Multi-agency Risk Assessment Conference
NRC: Non-randomised comparative study
PsycINFO: Psychological Information Database
QASCO: Questionnaire on Attitudes Consistent with Sexual Offending
RCT: randomised controlled trial
RoB2: Risk of Bias 2 tool
RSES: Rosenberg Self-Esteem Scale
SSCI: Social Sciences Citation Index
