# Supplementary material for: Effectiveness of UK-based support interventions and services aimed at adults who have experienced or used domestic and sexual violence and abuse: a systematic review and meta-analysis
Source: BMC Public Health. 2025 Mar 14;25:1003. doi: 10.1186/s12889-025-21891-5 (PMC11908015; doi:10.1186/s12889-025-21891-5)
Supplement: Supplementary file 3 — Additional file 3. Risk of bias assessments. Contains tables A1 and A2, detailing the risk of bias assessments for randomised controlled trials, non-randomised comparative trials and uncontrolled before and after studies (Table A1), and for the grey literature (Table A2). [file 12889_2025_21891_MOESM3_ESM.pdf]

Additional file 3 – Risk of bias assessments

Table A1. Risk of bias ratings for randomised controlled trials, non-randomised comparative trials and uncontrolled before and after studies

	Randomisation	Confounding	Selection of participants into the study	Classification of interventions	Deviations from intended interventions	Missing data	Measurement of the outcome	Selection of the reported result	Overall
**Randomised controlled trials (RoB2)**									
Gilchrist 2021	Low	-	-	-	Some concerns	High	High	Low	High
**Non-randomised comparative trials (Original ROBINS-I)**									
Halliwell 2019	-	Serious	Low	Low	No information	Serious	Serious	Serious	Serious
Smith 2015	-	Serious	Low	Low	No information	Serious	Serious	Serious	Serious
**Uncontrolled before and after studies (adapted ROBINS-I)**									
Calvert 2015	-	Serious	Low	Low	Moderate	Low	Low	Moderate	Serious
Clarke 1994	-	Serious	Low	Low	No information	Low	Low	Moderate	Serious
Clarke 2000	-	Serious	Low	Low	No information	Low	Low	Moderate	Serious
Ellis 2012	-	Serious	Low	Low	No information	Low	Low	Moderate	Serious
Karatzias 2016	-	Serious	Low	Low	No information	Serious	Low	Moderate	Serious
Howarth 2016	-	Serious	Low	Low	No information	Serious	Low	Moderate	Serious
Lindsay 2011	-	Serious	Low	Low	No information	Low	Low	Moderate	Serious
Murphy 2007	-	Serious	Low	Low	No information	Moderate	Low	Moderate	Serious
Rose 2012	-	Serious	Low	Low	No information	Low	Low	Moderate	Serious

Table A2. Risk of bias ratings for grey literature using the AACODS checklist

	**Authority**				**Accuracy**				**Coverage**				**Objectivity**				**Date**				**Significance**			
	**Y**	**N**	**NI**	**NA**	**Y**	**N**	**NI**	**NA**	**Y**	**N**	**NI**	**NA**	**Y**	**N**	**NI**	**NA**	**Y**	**N**	**NI**	**NA**	**Y**	**N**	**NI**	**NA**
**Bowen 2003**	5/9	1/9	2/9	0/9	5/8	2/8	0/8	1/8	1/1	0/1	0/1	0/1	2/2	0/2	0/2	0/2	2/3	0/3	0/3	1/3	5/7	1/7	1/7	0/7
**CAADA 2012**	3/9	0/9	6/9	0/9	5/8[1]	2/8	0/8	1/8	0/1	1/1	0/1	0/1	1/2	0/2	1/2	0/2	2/3	0/3	0/3	1/3	6/7[3]	1/7	0/7	0/7
**Geoghegan-Fittall 2020**	5/9	1/9	2/9	1/9	5/8[1]	2/8	0/8	1/8	0/1	1/1	0/1	0/1	2/2	0/2	0/2	0/2	2/3	0/3	0/3	1/3	5/7	1/7	1/7	0/7
**Howarth 2009**	6/9	1/9	0/9	2/9	5/8	2/8	0/8	1/8	1/1	0/1	0/1	0/1	2/2	0/2	0/2	0/2	2/3	0/3	0/3	1/3	7/7	0/7	0/7	0/7
**Ormston 2016**	3/9	0/9	3/9	3/9	5/8[1]	1/8	1/8	1/8	1/1	0/1	0/1	0/1	2/2	0/2	0/2	0/2	1/3	1/3	0/3	0/3	5/7	2/7		0/7
**Safelives (IDVA) 2013**	2/9	1/9	0/9	6/9	4/8[1]	2/8	1/8	1/8	0/1	1/1	0/1	0/1	0/2	0/2	0/2	2/2	1/3	0/3	0/3	2/3	7/7[2]	0/7	0/7	0/7
**Safelives (IDVA) 2014**	2/9	1/9	0/9	6/9	4/8[1]	2/8	1/8	1/8	0/1	1/1	0/1	0/1	0/2	0/2	0/2	2/2	1/3	0/3	0/3	2/3	7/7[2]	0/7	0/7	0/7
**Safelives (IDVA) 2017**	2/9	1/9	0/9	6/9	4/8[1]	2/8	1/8	1/8	0/1	1/1	0/1	0/1	0/2	0/2	0/2	2/2	1/3	0/3	0/3	2/3	7/7[2]	0/7	0/7	0/7
**Safelives (IDVA) 2019**	2/9	1/9	0/9	6/9	4/8[1]	2/8	1/8	1/8	0/1	1/1	0/1	0/1	0/2	0/2	0/2	2/2	1/3	0/3	0/3	2/3	7/7[2]	0/7	0/7	0/7
**Safelives (IDVA) 2021**	2/9	1/9	0/9	6/9	4/8[1]	2/8	1/8	1/8	0/1	1/1	0/1	0/1	0/2	0/2	0/2	2/2	1/3	0/3	0/3	2/3	7/7[2]	0/7	0/7	0/7
**Safelives (Outreach) 2013**	2/9	1/9	0/9	6/9	4/8[1]	2/8	1/8	1/8	0/1	1/1	0/1	0/1	0/2	0/2	0/2	2/2	1/3	0/3	0/3	2/3	7/7[2]	0/7	0/7	0/7
**Safelives (Outreach) 2014**	2/9	1/9	0/9	6/9	4/8[1]	2/8	1/8	1/8	0/1	1/1	0/1	0/1	0/2	0/2	0/2	2/2	1/3	0/3	0/3	2/3	7/7[2]	0/7	0/7	0/7
**Safelives (outreach) 2017**	2/9	1/9	0/9	6/9	4/8[1]	2/8	1/8	1/8	0/1	1/1	0/1	0/1	0/2	0/2	0/2	2/2	1/3	0/3	0/3	2/3	7/7[2]	0/7	0/7	0/7
**Safelives (outreach) 2019**	2/9	1/9	0/9	6/9	4/8[1]	2/8	1/8	1/8	0/1	1/1	0/1	0/1	0/2	0/2	0/2	2/2	1/3	0/3	0/3	2/3	7/7[2]	0/7	0/7	0/7
**Safelives (outreach) 2021**	2/9	1/9	0/9	6/9	4/8[1]	2/8	1/8	1/8	0/1	1/1	0/1	0/1	0/2	0/2	0/2	2/2	1/3	0/3	0/3	2/3	7/7[2]	0/7	0/7	0/7
**Taylor Dunn 2019**	6/9	3/9	0/9	0/9	5/8	2/8	0/8	1/8	0/1	1/1	0/1	0/1	2/2	0/2	0/2	0/2	2/3	0/3	0/3	1/3	5/7	1/7	1/7	0/7
**Webster 2015**	4/9	2/9	0/9	3/9	4/8	3/8	0/8	1/8	0/1	1/1	0/1	0/1	2/2	0/2	0/2	0/2	1/3[3]	0/3	0/3	1/3	5/7	1/7	1/7	0/7

Note: Y: Yes or probably yes; N: No or probably no; NI: No information or unclear; NA: Not applicable

[1] One or more items assessed as 'yes' but note that there is some detail lacking.

[2] One or more items assessed as 'yes' but note that significance is limited due to only data being presented and the context provided being limited.

[3] One or more items assessed as 'somewhat / a little'.
