# Supplementary material for: Effectiveness of UK-based support interventions and services aimed at adults who have experienced or used domestic and sexual violence and abuse: a systematic review and meta-analysis
Source: BMC Public Health. 2025 Mar 14;25:1003. doi: 10.1186/s12889-025-21891-5 (PMC11908015; doi:10.1186/s12889-025-21891-5)
Supplement: Supplementary file 4 — Additional file 4. GRADE Certainty Assessment. Contains Table A3, which details the assessments of certainty for each of the outcomes using the GRADE framework. [file 12889_2025_21891_MOESM4_ESM.pdf]

Additional file 4 – GRADE certainty assessment

Table A3: GRADE Certainty Assessment

Certainty assessment						
Participants (studies) Follow-up	Risk of bias	Inconsistency	Indirectness	Imprecision	Publication bias	Overall certainty of evidence
**Cessation of abuse (IDVA)**						
33708 (11 observational studies)	serious[a]	very serious[b]	not serious	not serious	none	⊕◯◯◯ Very low
**Cessation of abuse (Outreach)**						
8675 (5 observational studies)	serious[a]	very serious[b]	not serious	not serious	none	⊕◯◯◯ Very low
**Self-esteem (assessed with: Rosenberg Self-Esteem Scale)**						
467 (6 observational studies)	serious[a]	not serious	not serious	not serious	none	⊕⊕⊕◯ Moderate
**Attitudes to sexual offending (assessed with: Questionnaire on Attitudes Consistent with Sexual Offending)**						
35 (3 observational studies)	serious[a]	not serious	not serious	serious[c]	none	⊕⊕◯◯ Low
**Desirable responding (assessed with: Balanced Inventory of Desirable Reporting)**						
211 (3 observational studies)	serious[a]	serious[d]	not serious	serious[c]	none	⊕◯◯◯ Very low

Explanations

a. Downgraded once because the majority of the evidence had a crucial limitation for one criterion, or some limitations for multiple criteria, sufficient to lower confidence in the estimate of effect.
b. Downgraded twice because there was considerable heterogeneity (I2 >75%).
c. Downgraded once because the information size (the total number of participants) across all studies is less than the threshold of 400.
d. Downgraded once because the direction of effects across studies was not consistent.
