# Supplementary material for: Effectiveness of UK-based support interventions and services aimed at adults who have experienced or used domestic and sexual violence and abuse: a systematic review and meta-analysis
Source: BMC Public Health. 2025 Mar 14;25:1003. doi: 10.1186/s12889-025-21891-5 (PMC11908015; doi:10.1186/s12889-025-21891-5)

Additional file 5 – Sensitivity analyses (leave one out analysis)

Figure A1: Leave one out analysis of the Cessation of Abuse outcome for advocacy/IDVA interventions.

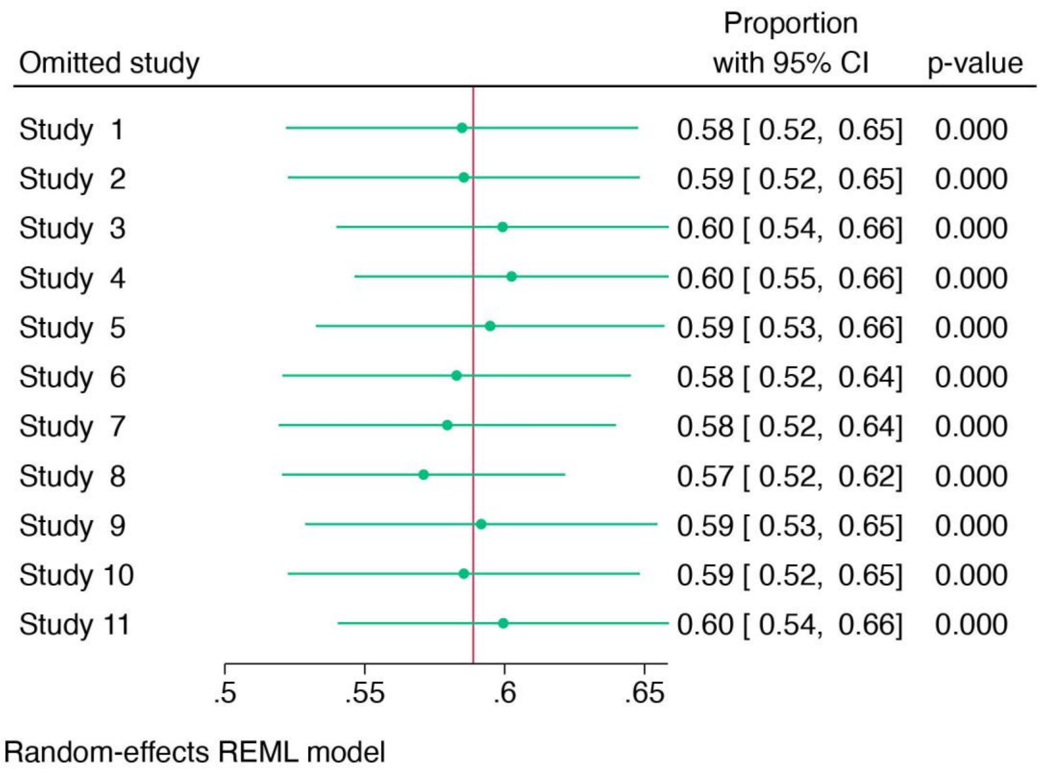

Figure A2: Leave one out analysis of the Cessation of Abuse outcome for outreach interventions.

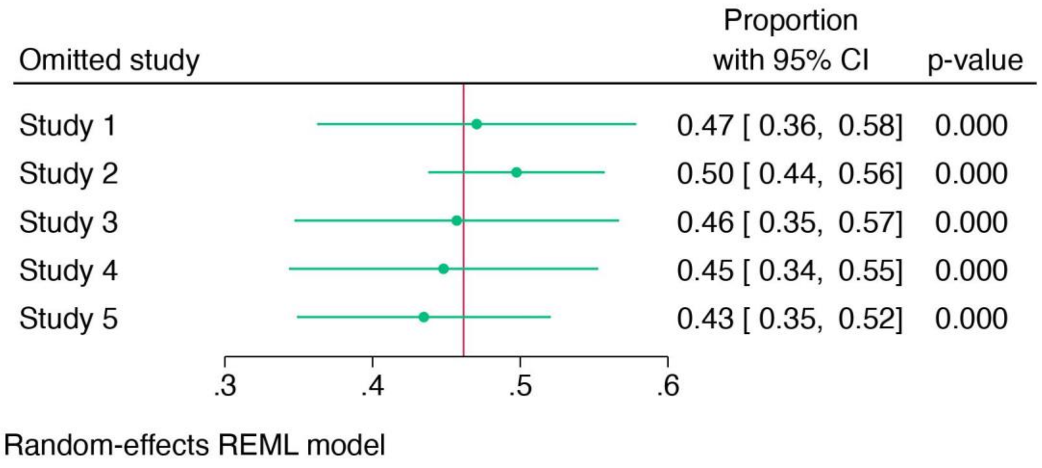

Supplement: Supplementary file 5 — Additional file 5. Sensitivity analyses (leave one out analysis). Contains Figures A1 and A2 which show the leave one out analyses for the Cessation of Abuse outcome for advocacy/IDVA interventions outcome, and the Cessation of Abuse outcome for outreach interventions outcome. [file 12889_2025_21891_MOESM5_ESM.pdf]
